# Myostatin and beyond in cirrhosis: all roads lead to sarcopenia

**DOI:** 10.1002/jcsm.12262

**Published:** 2017-11-23

**Authors:** Srinivasan Dasarathy

**Affiliations:** ^1^ Professor of Medicine, Cleveland Clinic Lerner College of Medicine; Director, Liver Metabolism Research; Staff, Departments of Gastroenterology Hepatology and Pathobiology, Cleveland Clinic Cleveland OH USA

**Keywords:** Liver, hyperammonemic stress response, proteostasis, ammonia, outcome

Sarcopenia or loss of skeletal muscle mass is a major complication of cirrhosis and liver disease.^1^, ^2^, ^3^, ^4^, ^5^, ^6^ A large body of literature exists to support the prognostic significance of sarcopenia in cirrhosis.^1^, ^2^, ^5^, ^7^ Independent clinical consequences of sarcopenia in cirrhosis include lower survival and quality of life, increases risk of complications including infections and encephalopathy, and lower post liver transplant survival.^1^, ^2^, ^3^, ^4^, ^5^, ^7^, ^8^, ^9^ Interestingly, unlike other complications of cirrhosis, sarcopenia does not reverse and usually worsens after liver transplantation^10^, ^11^, ^12^ that raises an important question of the utility of targeting sarcopenia for therapy before transplantation. Because the majority of patients with cirrhosis do not undergo transplantation and the window of opportunity is widest prior to transplantation,^7^ the focus should be in trying to reduce the severity and frequency of sarcopenia in cirrhosis prior to transplantation. Even though clinicians nearly universally recognize the high clinical significance of sarcopenia in cirrhosis, there are no effective treatment options.^3^, ^7^ The major reason for lack of effective therapies has generally been attributed to a limited understanding of the underlying mechanisms of sarcopenia in cirrhosis. However, other factors include the lack of precise measures of sarcopenia, absence of sensitive and specific biomarkers, and therapies that are based on deficiency replacement rather than mechanistic targets.^13^

The work by Nishikawa *et al*. in this issue goes towards addressing a number of these issues.^14^ In a very elegant study, the investigators quantified serum myostatin in a large cohort of patients with cirrhosis. Subjects were stratified by gender and median concentration of serum myostatin. Serum myostatin was significantly higher in males than females. Myostatin concentrations were higher with worsening severity of liver disease measured by Child Pugh score, a standard clinical method to predict outcomes in cirrhosis. Higher myostatin concentration was an independent predictor of worse survival in both male and female patients with cirrhosis. Finally, serum myostatin concentrations were associated with lower muscle mass measured as psoas muscle index on computed tomography, serum ammonia concentration, serum albumin, and branched chain to tyrosine ratios. These studies complement an earlier brief report that serum myostatin was elevated in cirrhosis, but one must note that circulating myostatin concentrations are elevated in heart failure and COPD also.^15^, ^16^ These studies are therefore of broad interest to investigators and physicians taking care of patients with other chronic diseases with sarcopenia. The present study also reiterates their data that circulating myostatin is inversely related to skeletal muscle mass but extends these data by demonstrating the prognostic significance and relate it to underlying pathophysiological perturbations. These investigators also report the use of psoas muscle index as a measure of muscle mass. This requires the use of imaging techniques while myostatin measurement is done in blood samples with lower costs and no risk of radiation exposure. Whether serial myostatin measurements will correlate with serial changes in muscle area or provide a better predictor of progressive muscle loss is an intriguing possibility because more rapid muscle loss worsens outcome in cirrhosis.^17^

Since the discovery of myostatin, the number of publications has increased exponentially with a detailed characterization of its biological properties.^18^, ^19^ Even though myostatin is consistently expressed in skeletal muscle, other tissues also express and possibly secrete myostatin.^18^ Signalling and functional responses to myostatin have focused on a paracrine effect even though there is increasing interest in myostatin as an endocrine factor or myokine. Consistent with its being a member of the TGFβ superfamily, myostatin binds to its receptor, activin IIB receptor, a type 2 transmembrane protein.^19^, ^20^, ^21^ Upon binding with myostatin, activin IIBR then heterodimerizes with a type 1 receptor, activin‐like kinase 4 or 5 in a context dependent manner, and this complex functions as a serine threonine kinase to phosphorylate Smad2/3 that in turn transcriptionally regulates target genes.^22^ Myostatin has also been reported to regulate a number of other signalling proteins and transcription factors including β catenin, forkhead box, and 5' adenosine monophosphate‐activated protein kinase.^23^, ^24^, ^25^ Unlike the extensive work done on the downstream signalling responses to myostatin, there is more limited data on the upstream regulation and the mechanisms of increased myostatin in disease states.^20^, ^24^, ^26^, ^27^, ^28^, ^29^, ^30^, ^31^, ^32^, ^33^, ^34^, ^35^, ^36^ A number of promoter analyses of myostatin in different species have been reported and targeted studies on specific transcription factors have been published.^20^, ^26^, ^32^, ^33^, ^37^, ^38^, ^39^ However, the mechanisms of context specificity have yet to be determined including the possible differential response to exogenous and endogenous myostatin.^28^ Most studies that have dissected the biological consequences of myostatin have used exogenous administration or delivery of myostatin with few studies on the response to endogenously stimulated myostatin, and these are of interest to not only translational scientist but also to clinical investigators because of the implications for therapy.

In addition to the novel observation by Nishikawa *et al*. that circulating myostatin was correlated with overall survival, another important observation was that these concentrations related to ammonia concentrations.^14^ Ammonia has been reported to transcriptionally upregulate myostatin via an NFkB dependent mechanism in the skeletal muscle but whether such a mechanism is relevant in other tissues is currently unknown.^40^ Ammonia is a cytotoxic molecule generated by a variety of physiological processes including amino acid and purine catabolism and gut microbial metabolism.^40^, ^41^ The hepatocyte is the only cell that is capable of metabolizing ammonia to urea, a relatively non‐toxic metabolite that is excreted by the kidneys. In liver disease, due to a combination of hepatocellular dysfunction and portosystemic shunting, circulating ammonia concentrations are increased, and the major clinical consequences are noted in the brain with the development of encephalopathy or coma. One protective mechanism is the skeletal muscle uptake of ammonia, and this was has been reported by three independent groups, but it was always believed that the skeletal muscle functioned as a metabolic sink and converted the ammonia to glutamine.^40^, ^42^, ^43^ Glutamine has cytoregulatory properties, and different cells use circulating glutamine as an anaplerotic substrate to regenerate ammonia that again needs to be removed by the hepatocytes.^44^, ^45^, ^46^, ^47^ Because ureagenesis is impaired in cirrhosis, there is no permanent disposal of ammonia, and the circulating glutamine serves as a source of ammoniagenesis in those tissues that utilize glutamine, maintaining hyperammonemia that is taken up again by the skeletal muscle. This pathway thus essentially transfers the carbon skeleton from the tricarboxylic acid (TCA) cycle in the muscle to other tissues resulting in skeletal muscle bioenergetics dysfunction and consequent impaired proteostasis and sarcopenia.

In the skeletal muscle, muscle, ammonia enters the myotubes, most likely by the RhBG class of ammonia transporters.^48^ Other perturbations in cirrhosis can also activate myostatin and include a reduction in growth hormone, testosterone, and increased tumour necrosis factor α. However, whether reversing these abnormalities can reverse myostatin is not known.^13^ In contrast, increased myostatin expression in response to hyperammonemia was reversed in response to ammonia withdrawal in myotubes *in vitro* or ammonia lowering measures in the portacaval anastomosis rat.^49^

**Figure 1:**
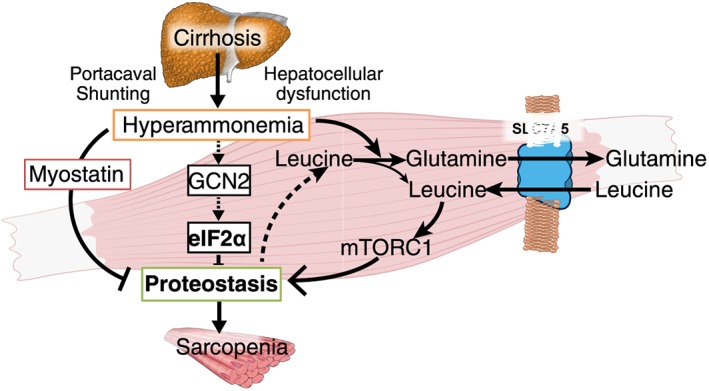
Of the various metabolic, hormonal and cytokine abnormalities in cirrhosis, hyperammonemia perturbs a number of signalling and molecular pathways. Myostatin is transcriptionally upregulated in the muscle that impairs mammalian target of rapamycin complex 1 signalling that decreases protein synthesis and increases autophagy. As a metabolic response, ammonia disposal occurs *via* glutamine synthesis that is in turn exchanged for leucine (and potentially other branched chain amino acid) that enter the muscle cell providing an explanation for decreased plasma branched chain amino acid in cirrhosis. An additional cellular response via the general control nondepressible 2‐eukaryotic initiation factor 2α axis impairs protein synthesis. There are a number of potential points of cross talk between these metabolic and molecular responses to hyperammonemia, all of which contribute to dysregulated proteostasis and sarcopenia.

In addition to the myostatin mediated signalling perturbations during hyperammonemia, ammonia is converted to glutamate in the mitochondria by cataplerosis of the critical TCA cycle intermediate, α ketoglutarate, and subsequent conversion of glutamate to glutamine in the skeletal muscle that is then exchanged for leucine by SLC7A5^47^, ^50^, ^51^ (Figure 1). These reactions can explain elevated circulating glutamine in cirrhosis. Both hyperammonemia and loss of α ketoglutarate contribute to the loss of muscle mass and mitochondrial dysfunction and reduced adenosine triphosphate content with impaired contractile function.^52^ Even though contractile function was not measured in these subjects, deconditioning or frailty is being increasingly recognized as an independent adverse prognostic indicator in cirrhosis.^53^, ^54^ Even though contractile function and muscle mass are not necessarily related, it is, however, possible that the underlying mechanisms that result in these clinical manifestations may be common including reduced bioenergetics as has been reported in the past.^52^, ^55^ Recent data also show post‐translational modifications of proteins may be responsible for impaired muscle strength and consequent frailty.^52^ This is important because even though myostatin depletion results in greater muscle mass, over time, muscle strength is not consistently maintained.^56^, ^57^, ^58^

Another interesting observation reported by Nishikawa is the relation between myostatin and serum albumin and tyrosine to branched chain amino acid (BCAA) ratios. Even though these have been considered as measures of ‘nutritional status’ in the past,^59^ it is increasingly recognized that the term ‘malnutrition’ in cirrhosis needs to be replaced by more specific terms.^7^ Two major components of ‘malnutrition’ in adult patients are being recognized: loss of skeletal muscle mass or sarcopenia and alteration in energy metabolism.^3^ Even though these seem disparate, in metabolic terms, these are interrelated. Sarcopenia was initially used by Rosenberg to refer to the progressive loss of skeletal muscle with weakness that occurs with aging.^60^ However, the term sarcopenia is translated to loss of skeletal muscle mass (sarcos, flesh; penia, deficiency) and is now used to refer to muscle loss in chronic diseases.^57^, ^61^ In contrast, serum albumin is believed to be a measure hepatocyte synthetic capacity. Current data supports the role of myostatin primarily in the skeletal muscle.^36^ However, albumin synthesis requires essential amino acids that are derived from dietary sources or endogenous proteolysis.^62^ However, since cirrhosis is a state of accelerated starvation,^63^ it is possible that the muscle protein synthesis is restricted to divert amino acids for synthesis of critical proteins including albumin in the hepatocytes. This hypothesis this needs to be explored in metabolic studies using tracer techniques.

The tyrosine to BCAA ratio is another measure that the authors have used as a measure of hepatic protein synthesis but is truly reflects the severity of liver disease and is due to skeletal muscle proteolysis and BCAA utilization.^47^, ^64^, ^65^ It is also recognized that BCAA are a metabolized primarily in the skeletal muscle as a source of energy and for potential detoxification of ammonia via anaplerotic influx into the TCA cycle (Figure 1).^47^, ^51^, ^65^ BCAA especially leucine and isoleucine can also function as a source of acetyl coenzyme A (CoA) independent of pyruvate because ammonia inhibits pyruvate dehydrogenase.^66^, ^67^, ^68^ These provide a mechanistic basis for low plasma BCAA in cirrhosis. Interestingly, L‐leucine also activates mammalian target of rapamycin complex 1 that increases protein synthesis and decreases autophagy that restores proteostasis or protein homeostasis and reverse sarcopenia.^51^, ^69^

In addition to myostatin dependent dysregulated proteostasis and sarcopenia, cellular stress pathways are activated during hyperammonemia.^51^ Unlike canonical stress pathways mediated *via* a number of eukaryotic initiation factor 2α kinases including general control non‐derepressed 2 that is activated in response to amino acid deficiency and during protein kinase R‐like endoplasmic reticulum kinase that is activated during unfolded or misfolded proteins.^70^, ^71^, ^72^ During hyperammonemia, a novel stress response has been reported that results in phosphorylation of the α subunit of the eukaryotic initiation factor with inhibition of protein synthesis.^51^ Even though hyperammonemia activates both myostatin and the HASR, the crosstalk between these pathways needs investigation (Figure 1).

**Figure 2:**
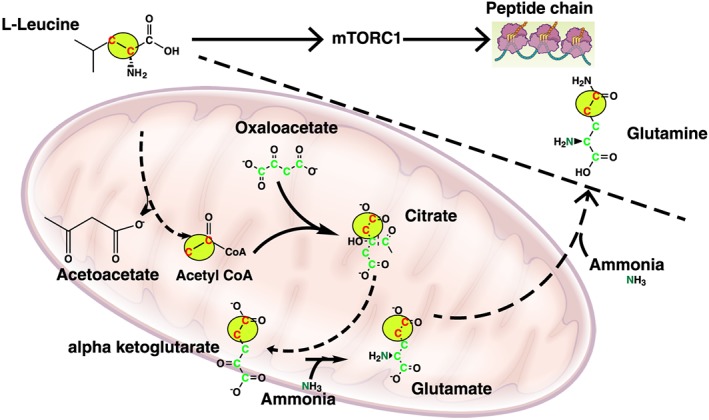
Leucine and potentially isoleucine and valine are selectively partitioned to the mitochondria to provide a source of acetyl coenzyme A as well as an anaplerotic substrate during hyperammonemia. This may explain the impaired mammalian target of rapamycin complex 1 signalling that is responsive to a high dose of leucine supplementation.

The implications of the report by Nishikawa *et al*. for developing treatment options cannot be overemphasized.^14^ Currently, the major approach to therapy in medicine is based on targeting deficiency rather than focusing on the mechanisms.^13^ Their report shows that myostatin and hyperammonemia are potential mechanistic treatment targets. Unfortunately, myostatin antagonists have not yet become clinically available and ammonia‐lowering therapies have been used in human subjects only to reverse hepatic encephalopathy, the best‐known consequence of hyperammonemia.^36^, ^49^ However, as mentioned above, preclinical data do support the use of long‐term ammonia lowering as a potential treatment option that should be evaluated in randomized trials with serum myostatin as a measure of therapeutic response. BCAA have been used to treat the consequences of hyperammonemia in cirrhosis with limited benefit. One potential reason may be the selective partitioning into the mitochondria to provide the carbon skeletons for anaplerosis as well as acetyl‐CoA as a TCA cycle substrate (Figure 2).^51^ These molecular and metabolic alterations formed the rationale for a high‐dose leucine supplementation to satisfy the mitochondrial metabolic demand during hyperammonemia so that leucine in the cytoplasm can activate mTORC1 to restore proteostasis. Data from preclinical and clinical studies have supported such a beneficial mechanism and hold potential for long‐term treatment with such supplements.^51^, ^73^ However, since leucine supplementation did not lower blood ammonia, myostatin expression was not altered but mTORC1, the direct target of leucine was activated with restoration of proteostasis.^73^ The reasons for the high significance of the study by Nishikawa *et al*. is that in addition to providing a compelling rationale for the use of serum myostatin as a potential biomarker for muscle loss and prognosis in cirrhosis, they also lay the foundation for the use of serial measurement of circulating myostatin as a potential strategy to evaluate response to interventions targeting sarcopenia in cirrhosis and possibly other chronic diseases. Currently, there are no non‐invasive circulating biomarkers to determine response to treatments to prevent or reverse sarcopenia in liver and chronic diseases and if serum myostatin is indeed such a marker, it will fill a longstanding need in the field of muscle loss.
